# Empirically Testing Explanation Preferences in Computational Argumentation

**DOI:** 10.1007/s12559-026-10654-y

**Published:** 2026-09-12

**Authors:** Roos Scheffers, Floris Bex, Matthieu Brinkhuis

**Affiliations:** 1 Department of Information and Computing Sciences, Utrecht University, Princetonplein 5, 3584 CC Utrecht, The Netherlands; 2 School of Law, Utrecht University, Janskerkhof 3, 3512 BK Utrecht, The Netherlands

**Keywords:** Computational argumentation, Explanation, Logic, Argumentation, Cognitive science, Explainable artificial intelligence (XAI)

## Abstract

Computational argumentation is an AI approach for creating human-understandable systems and explanations. Within the computational argumentation literature, explanation definitions based on principles from cognitive science have been created to provide *selective* explanations, that is, to reduce the information included in explanations. However, it remains largely untested whether these explanations align with realistic explanation behaviour. In this study, we test and validate different explanation definitions based on computational argumentation. This is done through an experiment in which participants provide explanations for arguments by choosing from other presented arguments. Responses by participants are compared to three types of explanations based on the literature. This comparison leads us to conclude that people prefer short explanations consisting of related arguments. While these preferences partly align with argumentation-based explanation definitions, we also see that explanations are frequently shorter than those generated using these definitions. The results show that definitions from computational argumentation are indeed capable of providing human-like explanations, but that they could be further improved by creating explanation definitions that are even more selective.

**Supplementary Information:**

The online version contains supplementary material available at https://doi.org/10.1007/s12559-026-10654-y.

## Introduction

Computational argumentation is an important subfield of (explainable) artificial intelligence (XAI) [1, 2], in which concepts such as support and attack between arguments are captured in formal argumentation models. Computational argumentation has been used for XAI in three main ways. First, a stand-alone argumentation model can be used to both infer conclusions and provide explanations [3–7] using existing data such as movie reviews [8], expert knowledge from professionals like police investigators [3], or requirements specified by users [9]. Second, argumentation models can be used to explain other, less interpretable AI models, where the argumentation model helps the user to understand how the original model infers conclusions [10–12]. This approach has been used to explain, for example, Bayesian networks [11] and various (deep) machine learning models [12, 13]. A third, hybrid XAI approach has also emerged in which argumentation models and other (often less interpretable machine learning models) are combined. For example, [10] use argumentation to impose domain-specific constraints on a machine learning model, [14] learn preferences over arguments for actions using reinforcement learning, and [15] combine human and large language model annotations to create argument maps.

Computational argumentation is inspired by the argumentative nature of human reasoning [16, 17]. People naturally look for arguments to support conclusions and evaluate arguments put forward by others [18]. People also mentally prepare arguments in anticipation of having to defend their beliefs [19]. Concepts within formal argumentation, such as one argument defending another argument against counterarguments, thus ‘reinstating’ the original argument, have been shown in experiments with human reasoners to be cognitively plausible [20]. Moreover, human explanations are often contrastive, focusing on why one outcome occurred rather than another [21], a pattern that fits well with the argument-counterargument structure of computational argumentation [22]. Additionally, computational argumentation is non-monotonic, meaning that the acceptance status of an argument can change when new information is added. This is in contrast to monotonic approaches in logic, which assign static truth values. Non-monotonicity allows argument acceptance to change when new information appears, reflecting the dynamic, changing, and flexible nature of human reasoning [23]. Computational argumentation captures all of these elements of human reasoning, while at the same time maintaining the formal rigour of a computational approach.

Despite computational argumentation incorporating elements of human reasoning, (the output of) large argumentation models can quickly become complex and less interpretable as model size grows. To address this, several definitions of explanations in computational argumentation have been proposed [24–32]. These explanation definitions are inspired by ideas from cognitive science [21] to be *selective*, in that they select from the full argumentation model the information that is most relevant to the conclusion or claim that is to be explained. For example, Fan and Toni [24] propose a definition where only arguments related to the argument being explained (in terms of defending arguments) are included in an explanation, because other, unrelated arguments in the model do not provide useful information. Borg and Bex [32] introduce further selectiveness criteria for argumentative explanations – for example, providing explanations consisting of only the set of arguments that are *necessary or sufficient* to defend another argument, or providing the smallest possible explanation in terms of the number of defending arguments.

The explanation definitions in computational argumentation discussed above have been created to reduce the information included in argumentative explanations. While they are inspired by ideas from cognitive science, they provide a largely normative perspective, that is, what is a ‘correct’ selective explanation given a formal model of argumentation? As of yet, it remains unclear whether the explanations provided by these definitions align with how humans explain claims or arguments. Hence, to ensure argumentation-based explanations are both effective and cognitively plausible, they need to be tested empirically. Empirically testing explanations from computational argumentation is situated in the wider context of research into the interaction between logic and cognitive science, where logic can generate hypotheses about human reasoning that cognitive science can test empirically. One well-known example is the Wason selection task [33], designed to test whether people reason according to classical logical implication.

Whereas several studies have investigated the cognitive plausibility of different concepts in computational argumentation (e.g. reinstatement) [20, 34, 35], only one study has considered argumentation-based explanations [36]. In this study, it was found that people prefer arguments directly related to the topic of an explanation (i.e., defenders of the topic argument) to arguments that are indirectly related (i.e., defenders of defenders of the topic argument). This is one way in which people select arguments to include in an explanation. However, this does not give insight into how people select between multiple arguments that are all directly related to the topic of an explanation. Among such arguments, different explanation definitions in computational argumentation make varying choices about which to include (e.g., only the minimal amount [24] or only the sufficient arguments [32]).

To better understand how argumentation-based explanation definitions compare to human reasoning, we test and validate several explanation definitions from the computational argumentation literature, focusing on their ecological validity, that is, the extent to which these definitions align with human-generated explanations [37]. This is done through an experiment in which participants provide explanations for arguments. In the experiment, participants were presented with one argument they could assume to be true (the topic argument), several counterarguments to the topic argument, and several arguments defending the topic argument. Participants were then asked to explain the conclusion of the topic argument, given the counterarguments, by using the defending arguments. Responses by participants were compared to three types of explanation definitions from the computational argumentation literature [24, 32]. From these comparisons, we draw conclusions about human explanation preferences and demonstrate that explanation definitions based on argumentation largely align with these preferences.

The rest of this paper is structured as follows. In “Background”, we will introduce abstract argumentation frameworks, the computational argumentation models that are used in the experiments, focusing on specific explanation definitions for selective (i.e., sufficient, minimal, and compact) explanations. The “Related Work” section discusses the relevant theoretical framework, touching on experiments into reasoning and cognitive science and experiments into computational argumentation. The “Methods” section will introduce the hypotheses of this study, describe how the used argumentation frameworks were created, and describe how data was collected. Then, the “Results” section describes the explanations given by participants, how they compare to sufficient minimal, and compact explanations. Finally, the “Discussion” discusses the results and their wider implication for explanations based on formal definitions that are cognitively plausible.

## Background

This section will first introduce an example argumentative scenario and will then use it to introduce the relevant elements of computational argumentation, including different types of explanation definitions. These explanation types are used in the experiment introduced in Section “Methods” to formulate hypotheses about participant behaviour.

In Example 1.1, an example scenario consisting of eight arguments is introduced. Every argument consists of a premise that introduces new information and a conclusion based on this information. The topic argument *A* is the central argument that the scenario revolves around, and is assumed to be accepted (see Definition 1.1). Following the topic argument are two counterarguments that attack the topic argument. Next, there are three arguments that defend the topic argument by attacking the counterarguments; argument *E* attacks the first counterargument *B*, *G* attacks the second counterargument *C*, and *F* attacks both counterarguments *B* and *C*. Finally, there is one unrelated argument *I*, while it is in the same context, has no relation to the other arguments in this scenario (i.e., it does not attack or is attacked by any of the arguments *A* to *G*).

### Example 1.1

*Topic (A):* Jessie saw Stephen at the crime scene yesterday at the time of the crime. Therefore, Stephen is guilty.

*Counterarguments:*

(B) Stephen’s father testifies that Stephen was having dinner with his family at the time of the crime. Therefore, Stephen couldn’t have been seen at the crime scene at the time of the crime.
(C) Stephen testifies that he has never been to the crime scene. Therefore, Stephen couldn’t have been seen at the crime scene at the time of the crime.

*Other arguments:*

(E) Stephen’s father can be very forgetful sometimes, so he might have misremembered having dinner with Stephen.
(F) Neither Stephen nor his father are reliable witnesses on Stephen’s actions; therefore, their testimonies cannot be trusted.
(G) There is a picture of Stephen at the crime scene two years ago, so Stephen is lying when he says he’s never been at the crime scene.
(I) Jessie’s credit card was used to make a purchase near the crime scene around the time of the crime. Therefore, it is likely Jessie was at the crime scene when the crime occurred.

**Fig. 1:**
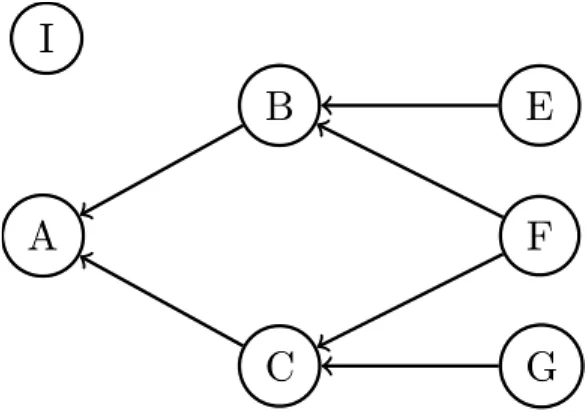
**A graphical representation of argumentation framework **$\mathcal{A}\mathcal{F}_1$ . This AF corresponds to the scenario in Example 1.1, where the topic argument is *A*, and the first and second counterarguments are arguments *B* and *C* respectively. The entire AF can be written as: $\mathcal{A}\mathcal{F}_1 = (\mathcal {A}_1,\mathcal {R}_1 )$, where $\mathcal {A}_1 = \{A,B,C,E,F,G,I\}$ and $\mathcal {R}_1 = \{(B,A), (C,A), (E,B), (F,B), (F,C), (G,C)\}$. Argument *I* is unrelated to the other arguments in the AF because there is no attack relation between *I* and any other argument

Arguments such as the ones in Example 1.1 can be represented as an *argumentation framework (AF)*, which is often used as the basic structure in computational argumentation [16]. An AF is a pair $\mathcal{A}\mathcal{F}= (\mathcal {A}, \mathcal {R})$, where $\mathcal {A}$ is a set of arguments, and $\mathcal {R}$ is a binary attack relation on these arguments, where an argument *X* attacks an argument *Y* iff $(X,Y) \in \mathcal {R}$. An argument *Z* defends argument *Y* if there is some $X \in \mathcal {A}$ such that $(X,Y) \in \mathcal {R}$ and $(Z,X) \in \mathcal {R}$, that is, *Z* attacks an attacker of *Y*. AFs can be represented as a directed graph, where the nodes represent arguments and the arrows represent the attack relations (Fig. 1). In this graph, the two arguments (*B*, *C*) attacking the topic argument (*A*) have direct arrows to the topic; this represents the attack relation (*B*, *A*) and (*C*, *A*). The three arguments that attack *B* and *C* defend argument *A* since they attack the attackers of *A*. The entire AF for the example is $\mathcal{A}\mathcal{F}_1 = (\mathcal {A}_1, \mathcal {R}_1 )$, where $\mathcal {A}_1 = \{A,B,C,E,F,G,I\}$ and $\mathcal {R}_1 = \{(B,A), (C,A), (E,B), (F,B), (F,C), (G,C)\}$

AFs can be used to draw conclusions based on conflicting information by evaluating arguments to determine sets of arguments that are collectively *acceptable* [16]. An argument *A* is acceptable with respect to a set of arguments *S* if every argument *B* that attacks *A* is attacked by *S*, that is, if there is a defender in *S* for every attacker of *A*. A set of acceptable arguments can still contain conflicting arguments; an *admissible* set is both conflict-free and defends all its members.

### Definition 1.1

Let $\mathcal{A}\mathcal{F}= (\mathcal {A}, \mathcal {R})$, $A \in \mathcal {A}$, and a set of arguments $S \subseteq \mathcal {A}$. Then:

- *A* is *acceptable* with respect to *S* iff each argument attacking *A* is attacked by any *B ∈ S*;
- *S* is *conflict-free* iff there exists no $(A,B) \in \mathcal {R}$ with *A, B ∈ S*; and
- *S* is *admissible* iff it is conflict-free and every $A \in \mathcal {A}$ is acceptable w.r.t. *S*.

If an argument $A \in \mathcal {A}$ is a member of an admissible set, we say it is *admissibly accepted*.

### Example 1.2

In $\mathcal{A}\mathcal{F}_1$, we are interested in the status of our topic argument *A*. Any set of arguments that defends *A* by attacking both *B* and *C* ensures *A* is acceptable. For such a set to also be admissible, it should be conflict-free, and every argument should be acceptable, therefore, an admissible set including *A* cannot include *B* or *C*, and has to include at least *F*, or both *E* and *G*. Two examples of admissible sets including *A* are $\{A,F\}$ and $\{A,E,F,G,I\}$.

Explaining a topic argument involves explaining why this topic argument is accepted, given the argumentation framework. While any admissible set containing the topic argument can provide information on why the topic was accepted, such a set may include arguments that do not have explanatory relevance, because they have no connection to the topic argument, such as argument *I* in Example 1.1. It is for this reason that [24, 32] define argumentative explanations as specific sets of arguments that contain only arguments that are relevant for the acceptance of the topic argument. As our formal framework is based on AFs with just attacks, we base relevance on the concept of defence.

### Definition 1.2

(Defence). Let $\mathcal{A}\mathcal{F}= (\mathcal {A}, \mathcal {R})$ and let $A, B \in \mathcal {A}$. Argument *Bdefends* argument *A* iff:

- *B* attacks *C* and *C* attacks *A* (direct defence); or
- *B* (directly or indirectly) defends *C* and *C* defends *A* (indirect defence).

A set $S \subseteq \mathcal {A}$ defends *A* if all arguments in *S* defend *A*. A set $S \subseteq \mathcal {A}$ defends a set $S' \subseteq \mathcal {A}$ if *S* defends all arguments in *S'*.

Thus, an argument *A* has explanatory relevance for the acceptability of another argument *A'* if *A* (directly or indirectly) defends *A*. For example, argument *I* in Example 1.1 is not relevant when explaining the acceptance of *A* since it does not attack any of *A*’s attackers, while *E*, *F*, and *G* are relevant because they attack *A*’s attackers *B* and/or *C*. Note that in all examples and scenarios of our study, there are only relevant arguments that directly defend the topic argument (as opposed to the scenarios in [36], where there were also scenarios in which the defender was attacked and, in turn, defended, thus creating an indirect defender for the topic argument).

Given the concept of defence, we can now define an explanation, that is, any set of arguments that is relevant for the acceptance of the topic argument.

### Definition 1.3

(Explanation) Let $\mathcal{A}\mathcal{F}= (\mathcal {A}, \mathcal {R})$, $A \in \mathcal {A}$, and $S \subseteq \mathcal {A}$. Set *S* is an explanation for topic argument *A* iff *S* defends *A*.

An explanation is thus a (possibly singleton) set of arguments that is relevant for the topic argument because it defends the topic argument against at least one attacker. Note that an explanation does not guarantee the topic argument is accepted, for that we need to define a *sufficient* explanation: a set of defending arguments is sufficient to explain an admissibly accepted topic argument if no other arguments are needed for the topic argument to be accepted [32].

### Definition 1.4

(Sufficient explanation). Let $\mathcal{A}\mathcal{F}= (\mathcal {A}, \mathcal {R})$ and let $A \in \mathcal {A}$ be admissibly accepted. The explanation $S \subseteq \mathcal {A}$ is *sufficient* for the acceptance of *A* if *S* (directly or indirectly) defends *A*, *S* is conflict-free, and *S* defends $S \cup \{A\}$ against all of its attackers.

### Example 1.3

In $\mathcal{A}\mathcal{F}_1$ in Fig. 1, there are only five possible sufficient explanations, since sufficient explanations cannot include *I*, which does not defend *A*. The sufficient explanations for *A* in $\mathcal{A}\mathcal{F}_1$ are $\{E,F,G\}$, $\{E,F\}$, $\{E,G\}$, $\{F,G\}$, and $\{F\}$.

So, sufficient explanations ensure that all arguments in the explanation are relevant for the acceptance of the topic argument because they all defend the topic argument. To be even more selective in the information included in an explanation, *compact* [38] and *minimal* [32] explanations have been defined. An explanation is *minimal* if it is the smallest possible sufficient explanation in terms of the number of arguments. An explanation is *compact* if it is the smallest possible sufficient explanation in terms of subset minimality, that is, there is no explanation that contains a subset of the arguments that is also an explanation.

### Definition 1.5

(Minimality and compactness) Let $\mathcal{A}\mathcal{F}= (\mathcal {A}, \mathcal {R})$, $A \in \mathcal {A}$, and let $A \in \mathcal {A}$ be admissible. Then:

- *S* is a *minimal* explanation for the acceptance of *A* iff S is a sufficient explanation for *A* and there is no sufficient explanation *S'* for *A* such that *|S'| < |S|*;
- *S* is a *compact* explanation for the acceptance of *A* iff S is a sufficient explanation for *A* and there is no sufficient explanation *S'* for *A* such that *S' ⊂ S*.

### Example 1.4

In $\mathcal{A}\mathcal{F}_1$ in Fig. 1, there are two possible compact explanations. These are $\{E,G\}$ and $\{F\}$, since neither of these explanations has a subset that is also a sufficient explanation. Since $\{G\}$ contains fewer arguments than any other sufficient explanation, it is the minimal explanation for this AF.

Table 1 shows all the possible combinations of ‘other arguments’ from Example 1.1 (these are all the possible explanations participants can choose for $\mathcal{A}\mathcal{F}_1$, cf. Section “Methods”). It shows how minimality, compactness, and sufficiency relate: every minimal explanation is compact and sufficient, and every compact explanation is sufficient. For an explanation to be sufficient, but not minimal or compact, there has to be an explanation that is smaller in size or an explanation that is a subset of the explanation, respectively. For an explanation to be sufficient and compact but not minimal, there has to be an explanation that is smaller in size, that is not a subset of the explanation.

**Table 1:** All possible sets consisting of the defending arguments and unrelated arguments in $\mathcal{A}\mathcal{F}_1$

Set	Minimal	Compact	Sufficient
E			
F	x	x	x
G			
I			
EG		x	x
EF			x
FG			x
FI			
EI			
GI			
EFG			x
EGI			
EFI			
FGI			
EFGI			

Note. The crosses indicate whether a set fits the explanation type for *A*. Out of the 15 possible explanations in $\mathcal{A}\mathcal{F}_1$, five are a sufficient explanation for *A*, two are a compact explanation for *A*, and one is the minimal explanation for *A*. The other nine sets do not fit any of the three types of explanation definitions. Minimal explanations are compact and sufficient, and compact explanations are also minimal

**Fig. 2:**
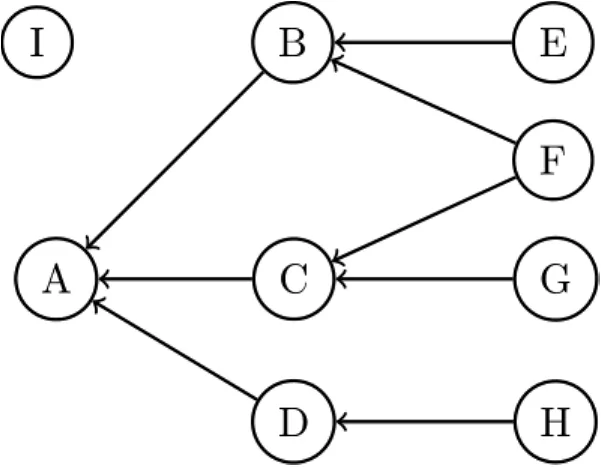
**A graphical representation of argumentation framework **$\mathcal{A}\mathcal{F}_2$**.** This AF corresponds to the scenario in Example 1.5, where the topic argument is *A*, and the counterarguments to *A* are arguments *B*, *C*, and *D*. The entire AF can be written as: $\mathcal{A}\mathcal{F}_2 = (\mathcal {A}_2,\mathcal {R}_2 )$, where $\mathcal {A}_2 = \{A,B,C,D,E,F,G,H,I\}$ and $\mathcal {R}_1 = \{(B,A), (C,A), (D,A), (E,B), (F,B), (F,C), (G,C), (H,D)\}$. In this AF, $\{E,G,H\}$ and $\{F,H\}$ are compact explanations, but only $\{F,H\}$ is minimal. Here $\{E,F,G,H\}$, $\{E,G,H\}$ and $\{F,H\}$ are sufficient explanations

In this experiment reported in this paper, we used two AFs. $\mathcal{A}\mathcal{F}_1$ is the smallest AF that allows for distinguishing between the minimal, compact, and sufficient explanations. We also used a second, larger AF, $\mathcal{A}\mathcal{F}_2$, (Fig. 2). This AF includes two additional arguments, *D* and *H*, which attack and defend the topic argument, respectively. See Examples Because of the addition of *H* that can be included in explanations for *A*, the total number of possible explanations that participants can give also increases from 15 in $\mathcal{A}\mathcal{F}_1$ to 30 in $\mathcal{A}\mathcal{F}_2$. The number of minimal, compact, and sufficient explanations for $\mathcal{A}\mathcal{F}_2$ is the same as for $\mathcal{A}\mathcal{F}_1$. In fact, since *A* now has to be defended against *D* in explanations, we obtain the minimal, compact, and sufficient explanation for $\mathcal{A}\mathcal{F}_2$ by adding *H* to the explanations fitting our three types for $\mathcal{A}\mathcal{F}_1$. The sufficient explanations for *A* in $\mathcal{A}\mathcal{F}_2$ are $\{E,F,G,H\}$, $\{E,F,H\}$, $\{E,G,H\}$, $\{F,G,H\}$, and $\{F,H\}$ The compact explanations are $\{E,G,H\}$ and $\{F,H\}$. The minimal explanation is $\{F,H\}$.

### Example 1.5

*Topic (A):* Reviewer 1 says that the paper makes an important contribution to the field. So, the paper should be accepted.

*Counterarguments:*

(B) Reviewer 2 says that the paper does not have enough experimental proof. So, the paper should be rejected.
(C) Reviewer 3 argues that the paper’s theory has problems. So, the paper should be rejected.
(D) The paper does not follow the journal’s style, showing a lack of care. So, the paper should be rejected.

*Other arguments:*

(E) Reviewer 2 is known to be very critical. So, their negative review is biased.
(F) The paper has an appendix with detailed experimental proof and an explanation of the theory. So, these parts are not lacking or flawed.
(G) Reviewer 3 often publishes work that challenges other theories. So, their review is biased by their research.
(H) Papers do not need to follow the journal’s style during the review. So, the style does not show a lack of care.
(I) The journal editor shared new rules for submitting papers to improve the review process. So, future reviews will be faster.

## Related Work

This study is situated in the wider context of research into the interaction between logic and cognitive science. Human reasoning is a topic in both of these fields. Logic examines reasoning from a normative perspective, aiming to define principles of correct reasoning by relying on intuitions and examples, as seen in classical and propositional logic. Cognitive science takes a descriptive approach, studying realistic human reasoning, including their cognitive limitations, biases, and errors. This has produced theories such as cognitive load theory [39], which can explain why people often diverge from formal logic under realistic conditions. Despite these different perspectives on reasoning, the two fields have long influenced each other [40, 41]. Formal logic can generate hypotheses about reasoning that cognitive scientists can test empirically to investigate reasoning. One well-known example is the Wason selection task [33], designed to test whether people reason according to classical implication. Cognitive science has also influenced developments in logic. Traditional formal logic, such as classical and propositional logic, assumes that reasoning follows strict rules of inference. However, cognitive science has shown that human reasoning is often influenced by factors such as context, cognitive limitations, and social interaction. As a result, logic has gradually incorporated more cognitive elements such as preferences, goals, and intentions, leading to richer logical theories that better reflect human reasoning, such as computational argumentation.

While computational argumentation is inspired by human argumentation, only a few studies have empirically investigated computational argumentation [42]. Of those studies, most have focused on how humans evaluate and interpret arguments and attacks between them, rather than on how humans explain arguments or claims. Since there is so little prior work on empirically investigating explanations in argumentation, this study incorporates methods and insights from broader empirical research in argumentation. Those studies are presented in the remainder of this section.

The first major experiment cognitively investigating computational argumentation was performed by Rahwan et al. [20], who tested how humans evaluate reinstatement. Recall that an argument is reinstated if it is attacked by an argument and then shown to be defended by another argument. In computational argumentation, a reinstated argument is equally acceptable as an unattacked argument. In their study Rahwan et al. [43] found that for participants, their confidence in an argument was highest when it was not attacked, was lowered when the argument was attacked, and went back up (but not fully restored) when the argument was reinstated.

In [34], the cognitive plausibility of various argumentation semantics was tested using two experiments. Argumentation semantics specify how to determine which sets of arguments can be considered acceptable, based on their attack relations. In the first experiment, participants were, in groups, tasked with drawing all attack relations between arguments in a set. With this experiment, they validated that participants correctly identified attack relations between arguments in a given AF. They found that conflicting arguments can be created that people interpret as unidirectional attacks, especially undercutting the trustworthiness of a source is convincing as a unidirectional attack. This provides valuable insights into how to instantiate AFs. In the second experiment, participants individually judged the acceptability of arguments and then discussed their answers in a group before making a final judgement. The researchers compared the acceptability of arguments as rated by participants to their acceptability under various argumentation semantics [16], and found that accuracies increased after group deliberation. They also found that semantics that minimize being undecided on the acceptance of arguments were most comparable to participant judgements.

In [44], people’s argument preferences were shown to vary across different domains. Participants were presented with natural language arguments from four different contexts and were tasked with indicating their agreement with the conclusions of the arguments. Participants also indicated why they chose this option and how relevant other arguments were. They found that preference between arguments is domain-dependent, and participants justify their choice with domain-specific reasons, such as *“All weather forecasts are notoriously inaccurate”*. This highlights the importance of tailoring explanations to the specific context in which they are used.

Argument preferences were also investigated by Rosenfeld and Kraus [45]. In their experiment, participants were presented with existing conversations and tasked to choose an argument to respond to the conversation. They found the acceptance status of an argument was a poor predictor of participant responses, but that instead, responses could be predicted well using a relevance heuristic. This relevance heuristic used both the path length from the currently presented argument to the latest argument and from the current argument to the topic argument and provided a better prediction of human behaviour than any of the argumentation semantics in the study.

An experiment by Polberg and Hunter [46] also presented participants with argumentative dialogues. Participants indicated their agreement with the arguments presented in the dialogue. Then, probabilistic argumentation [47] was used to represent the extent to which an argument is believed or disbelieved. They found that participants interpret arguments and relations differently and that their prior knowledge can affect this. They also suggest that simple accept/reject/undecided labels fail to capture the nuances of human judgments, suggesting richer argumentation systems could better capture human judgment.

Beyond argumentation frameworks, empirical studies in XAI have examined the effects of explanation format [48], goals [49], and content [50] on user understanding and decision-making. Other work highlights the importance of contrastive and causal explanations, showing that humans prefer explanations that clarify why one outcome occurred rather than another [21, 51, 52]. These findings indicate that human explanation behavior is influenced by cognitive and contextual factors, supporting the need for experimental evaluation of explanation definitions.

Despite the growing body of empirical cognitive research on the interpretation and evaluation of arguments in computational argumentation, only one study, to our knowledge, has empirically examined how people explain arguments. Scheffers et al. [36] investigated two types of relatedness in argumentation-based explanations, directly and indirectly related arguments, which correspond to direct and indirect defence from Definition 1.2. Participants were presented with scenarios based on argumentation frameworks and asked to use the arguments in that scenario to explain a topic argument. It was found that participants could identify the defending arguments and use them to explain (the acceptability of) the topic argument. Furthermore, participants preferred to use just the direct defender as an explanation, even when an indirect defender was also available.

Taken together, these studies show some important steps taken towards understanding how humans reason and interact with computational argumentation. However, more empirical research is needed to determine which explanation definitions are best to explain the acceptance of arguments to people and to determine exactly what information people prefer to include in explanations. Using directly related arguments is one way in which people select arguments to include in an explanation [36]. However, this does not give insight into how people select between multiple arguments that are all directly related to the topic of an explanation. Among such arguments, different explanation definitions in computational argumentation make varying choices about which and how many to include. Therefore, this study will examine three different explanation definitions from computational argumentation by asking participants to explain arguments in several argumentative scenarios, and comparing their explanations to the expected explanation based on computational argumentation theory.

## Methods

The research question for this study is: *what arguments do people prefer in argumentation-based scenarios, and does this align with three explanation definitions from computational argumentation?* To test this research question, we used three increasing selective hypotheses.

1. *Participants prefer sufficient explanations.*
2. *Participants prefer compact explanations.*
3. *Participants prefer minimal explanations.*

Participants were provided with scenarios like the one in Example 1.1, and then asked to assume the topic argument to be true^1^ and select one or more of the (defending or unrelated) ‘other arguments’ to explain the topic argument. *‘Participants prefer X explanations’* in the hypotheses then means explanations fitting type X are selected significantly more frequently than would be expected if the participants were choosing randomly. This randomness is operationalised using two baselines: a naive baseline and a simulated baseline (in Table 2). The naive baseline assumes that each explanation has an equal probability of being selected by participants. This means that the probabilities that each type of explanation is selected are based solely on the proportion of explanations that fit that explanation type. For example, for $\mathcal{A}\mathcal{F}_1$ introduced in Fig. 1, there are 5 sufficient explanation sets and a total of 15 possible explanation sets (see Table 1); the naive baseline is therefore 0.33, representing that if all explanations are selected equally, a third of the selected explanations will be sufficient. This baseline is considered naive because it assumes that all explanations are equally likely to be selected, regardless of the number of arguments they contain. However, it is not given that participants are equally likely to select an explanation consisting of a single argument compared to one containing four arguments. To address this, the simulated baseline was weighted using the empirical distribution over explanation lengths observed in this study. Specifically, we used the observed distribution of the number of selected arguments per explanation to estimate the probability that participants would randomly select each explanation type. For example, for $\mathcal{A}\mathcal{F}_1$, there is only one possible minimal explanation, which only contains one argument. For this AF, 62 percent of explanations consisted of a single argument, and there are four possible explanations of one argument for this AF. Therefore, the probability of randomly selecting a minimal explanation for $\mathcal{A}\mathcal{F}1$ is 0.62 * 0.25, which equals 0.16; this value can be found in the corresponding cell in Table 2.

**Table 2:** Values for the two baselines used in this study to test the hypotheses

	AF	Total	Sufficient	Compact	Minimal
Counts	1	15	5	2	1
	2	31	5	2	1
Naive baseline	1	15	0.33	0.13	0.07
	2	31	0.16	0.06	0.03
Simulated baseline	1	15	0.32	0.20	0.16
	2	31	0.08	0.04	0.03

Note. This study uses a naive and a simulated baseline to measure if the studied types of explanation definitions are selected more frequently than they would be if participants selected answers randomly. The naive baseline is calculated by dividing the count of an explanation type by the total possible explanations. The simulated baseline is weighted based on the number of arguments included in explanations by participants

### Scenario Creation

To instantiate the two AFs introduced in this paper ($\mathcal{A}\mathcal{F}_1$ in Figs. 1 and $\mathcal{A}\mathcal{F}_2$ in 2), we created 20 scenarios set in various domains, such as a crime scene investigation, environmental policy, and school projects. Each scenario consists of nine arguments matching $\mathcal{A}\mathcal{F}_2$, by leaving out arguments *D* and *H*, they can also be used to instantiate $\mathcal{A}\mathcal{F}_1$. To create these scenarios, we followed a similar format and argument structure as used in prior work [20, 34, 53]. In every scenario, argument *I* is unrelated to the other arguments. When creating instantiations for this argument, we ensured that the argument was not trivially unrelated by setting it same context (see argument *I* in Example 1.1).

The design of the scenarios was done in two steps. First, we wrote scenarios that intentionally included variance in several dimensions. This decision reflects our aim to study explanation preferences in a general sense, rather than preferences in any specific context or for specific types of arguments. In abstract argumentation, an argument is an abstract item with no internal structure. These abstract arguments can be instantiated with many types of formal or informal arguments. Therefore, our scenarios cover a wide range of arguments and attack types (see S4 Appendix for more details). Second, we ensured the scenarios were easy to understand for participants. Language was standardized in every scenario to CERF B1 or B2 level [54], making the entire collection of argument sets is B2 level on average. This means that a fluent speaker should have no problem reading the arguments. On average, each scenario is 194 words long. Additionally, a small pilot with a think-aloud procedure was conducted to ensure the understandability of the arguments. This led to minor tweaks in the experiment instructions and clarification of ambiguous argument phrasing.

### Participants

No sensitive or personally identifiable information was collected from participants, and all data was collected anonymously. The study was approved by the Utrecht University Science-Geo Ethics Review Board. Before starting the experiment, participants provided informed consent through the digital experiment software (see S1 Appendix). We recruited 301 participants (M = 34.5, SD = 12.0) through Prolific on November 21 and 22, 2024; this number of participants was determined using a power analysis and preregistered [55]. Participants were paid £3 for partaking in the experiment. Participants took, on average, 16.4 minutes, meaning they were paid, on average, £10.76 per hour. Participants were required to be at least 18 years old, be fluent English speakers, and have at least completed secondary education. Most participants (248) completed at least an undergraduate education. The gender of participants was mixed (Female 156, Male 144, Other 1). Participants were from 42 different countries, with the largest groups being from South Africa (104), the UK (55), and the US (16). In total, five participants were rejected for completing the experiment in less than five minutes, as it is impossible to read all items in that time.

### Procedure

Prior to the start of data collection, the methods and hypotheses of the current study were preregistered [55]. Participants were invited to take part in the experiment through Prolific. On the platform, they received some basic information about the experiment, how long it would take (about 20 minutes), and how much they would be paid (£3). After deciding to partake in the experiment, they were directed to the experiment, where they provided consent for their data to be collected (see S1 Appendix). After providing consent, participants were presented with detailed instructions and an example (full layout in S2 Appendix).

Participants were then presented with eight out of twenty possible scenarios, four per AF, which were presented one by one in random order. Every scenario consists of a topic argument, arguments attacking the topic argument, and other arguments defending or unrelated to the topic argument. Participants were shown these arguments in a similar way to Example 1.1. At the top of the page, the topic argument was shown, then the ‘counterarguments’ (clearly marked as such). The defending and unrelated arguments were not labelled as such, but simply grouped below the counterarguments in random order, with checkboxes next to them. Participants were instructed to explain the topic argument using these arguments, where participants could select multiple arguments but needed to select at least one before they could progress to the next scenario. After selecting an explanation for eight scenarios, participants finished the experiment and were redirected to Prolific.

## Results

Before testing the hypotheses, we checked the quality of responses. All participants spent at least five minutes completing the experiment and selected varying explanations throughout the experiment. No learning or fatigue effects were found. Next, we tested the consistency across the different scenarios. Each AF was instantiated with 20 different scenarios. To test if there was a difference between responses to scenarios, possibly indicating issues with the interpretation of scenarios, we used Fisher’s exact test^2^ to test for an association between scenario and explanation selected by participants. Since there is a dependency between explanation options, we only included explanations of a single argument. We found a significant relation (*p* < .001) between explanation and scenario, with a moderate effect size^3^. Despite these group-level differences, we found no differences between explanations provided for individual scenarios when comparing them to each other or to the average response pattern, using Fisher’s exact test. Since no single scenario was significantly different from the overall response pattern or any other scenario, we aggregated responses by AF for further analysis.

In the aggregated data, we observed a difference in the types of explanations provided by participants between $\mathcal{A}\mathcal{F}_1$ and $\mathcal{A}\mathcal{F}_2$. For $\mathcal{A}\mathcal{F}_1$, participants select all three explanation types more frequently than for $\mathcal{A}\mathcal{F}_2$. This suggests a difference in the interpretation of the two AFs by participants; therefore, the two AFs will be analysed separately.

**Fig. 3:**
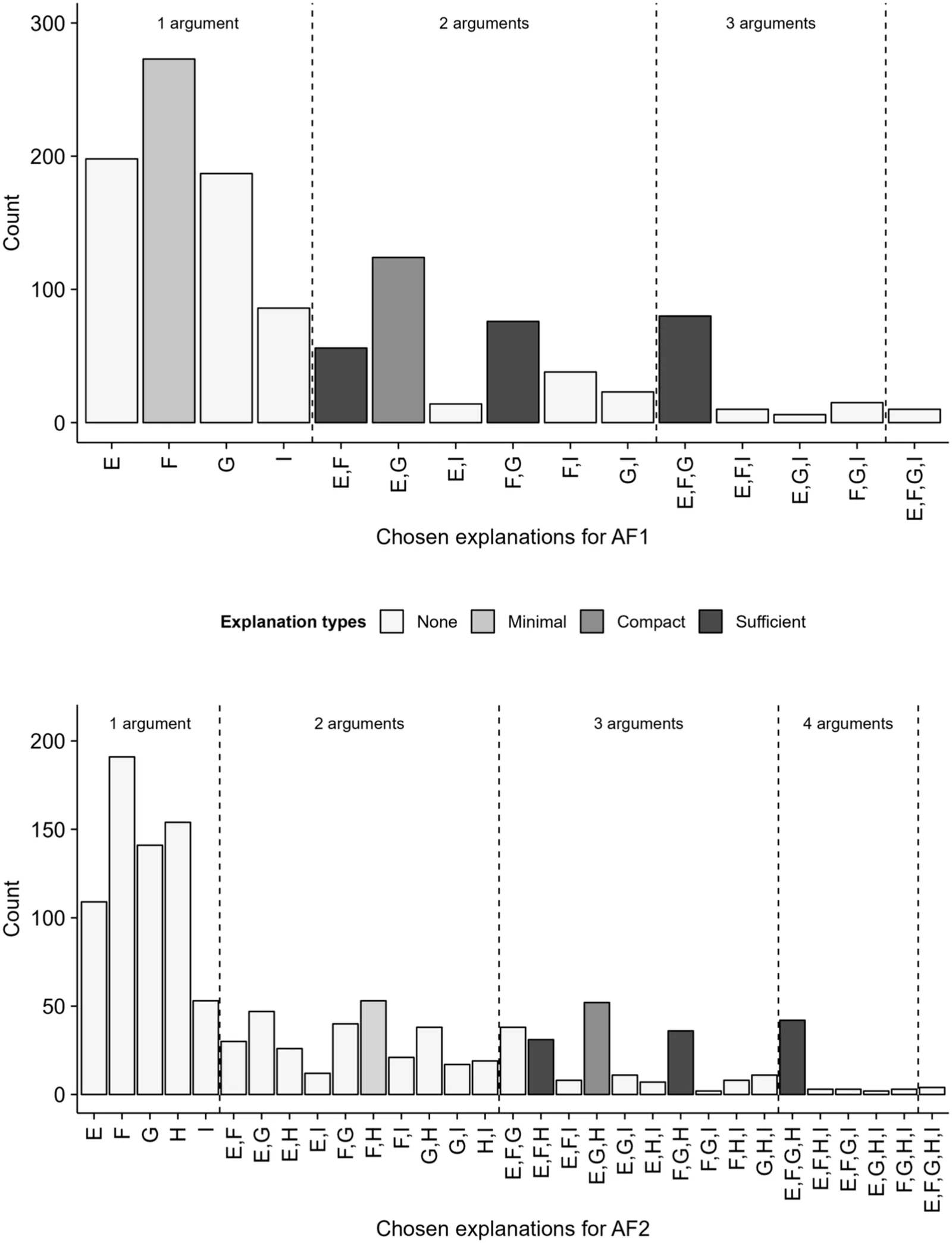
Counts for all explanations provided by participants for the two AFs. Note. There were 1196 total explanations for $AF_1$ (top) and 1212 for $AF_2$ (bottom). The bars are coloured by explanation type, and the dotted lines separate bar by the number of arguments included in explanations. Most explanations consist of a single argument, followed by explanations of two, three, four, and five arguments. For $AF_1$ (top), the minimal explanation is selected most frequently, and for $AF_2$ (bottom), the minimal explanation is the most frequently selected explanation of length two. For both AFs, the sufficient and compact explanations are among the most frequently selected within their respective explanation lengths. For $AF_2$, many explanations of a single argument are selected, which do not fit any of the explanation types considered in this study

### Main hypotheses

To test out hypotheses, we consider all explanations provided by participants for each of the two AFs (Fig. 3). The most frequent explanation in both AFs is $\{F\}$; this is a minimal explanation for $\mathcal{A}\mathcal{F}_1$, but not for $\mathcal{A}\mathcal{F}_2$, where the minimal explanation is $\{F,H\}$. After $\{F\}$, all other explanations containing one related argument are selected most frequently. Among explanations containing two arguments for $\mathcal{A}\mathcal{F}_1$, the compact ($\{E,G\}$) and sufficient ($\{E,F\}$, $\{F,G\}$) explanations were selected more frequently than other explanations containing three arguments. For $\mathcal{A}\mathcal{F}_2$, the minimal explanation $\{F,H\}$ was also selected more than any other explanation of two arguments. So, explanations fitting our hypothesised explanation types are selected more frequently than other explanations of the same length. A similar pattern can be observed for explanations of three and four arguments (Fig. 3). The only exception to this pattern is explanation $\{E,F,G\}$, which was selected frequently compared to other explanations of three arguments, despite not fitting any of the three explanations.

For $\mathcal{A}\mathcal{F}_1$, the average number of arguments included in explanations was 1.48 (SD = 0.54), 62 percent of explanations included one argument, 28 percent included two, 9 percent included three, and less than 1 percent included four arguments. For $\mathcal{A}\mathcal{F}_2$, the average length of explanations was 1.74 (SD = 0.76), which, using a Wilcoxon signed rank t-test^4^, was found to be significantly different (V = 3164, *p* < 0.001) from the explanation length for $\mathcal{A}\mathcal{F}_1$. For this $\mathcal{A}\mathcal{F}_2$, 53 percent of explanations included only one argument, 25 percent included two, 17 percent included three, and less than 5 percent of explanations included four or five arguments.

**Fig. 4:**
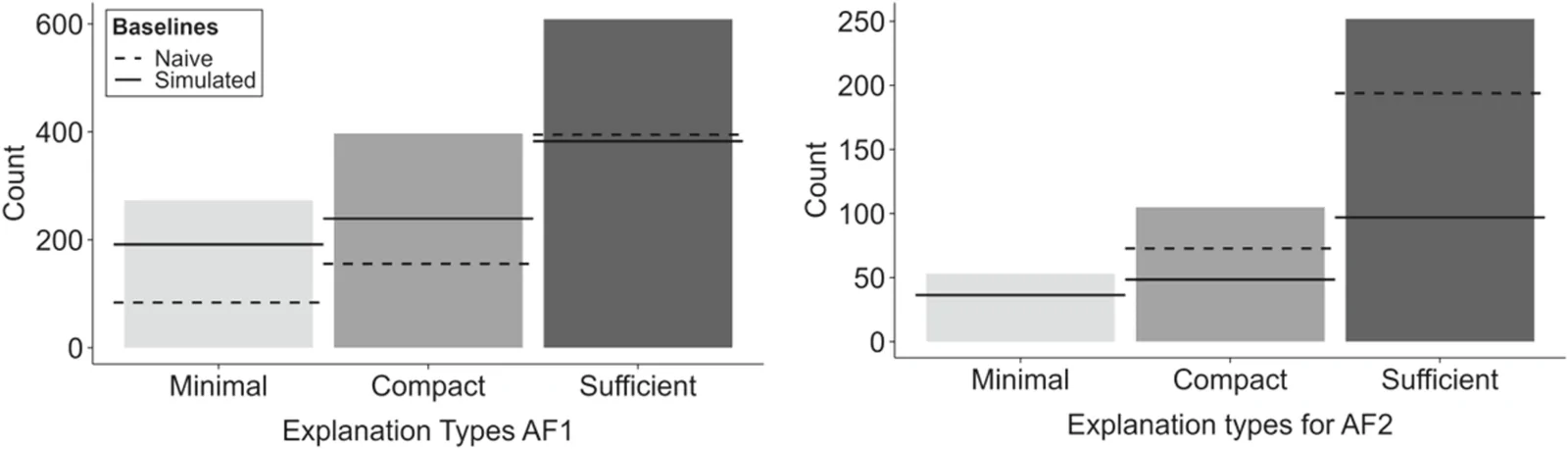
Counts of explanations grouped by explanation type for each of the two AFs. These counts match the coloured bars in Fig. 4; since all minimal explanations are also compact, and all compact explanations are also sufficient, these counts are cumulative. The dashed line indicates the naive baseline, the solid line indicates the simulated baseline, which is weighted using data on the explanation length of participants. Since participants generally gave short explanations, this baseline is higher for minimal and compact explanations in $\mathcal{A}\mathcal{F}_1$, which consist of one or two arguments, and lower for sufficient explanations in $\mathcal{A}\mathcal{F}_2$, which consist of up to four arguments.

When grouped, all explanation types are selected more frequently than expected based on both baselines (Fig. 4). For $\mathcal{A}\mathcal{F}_1$, a total of 1196 explanations were given. Of these total explanations, 609 out of 1196 ($\hat{p}$ = .509) explanations fit at least one of the three hypothesised explanation types. A total of 609 ($\hat{p}$ = .509) explanation were sufficient, 397 ($\hat{p}$ = .332) were compact, and 273 ($\hat{p}$ = .228) were minimal. These counts are compared to the baselines in Fig. 4. Since all minimal explanations are also compact, and all compact explanations are also sufficient, these counts are cumulative. The other 587 out of 1196 ($\hat{p}$ = .491) explanations did not fit any of the three types. These 587 explanations that did not fit any type mostly (471) consisted of a single argument.

For $\mathcal{A}\mathcal{F}_2$ a total of 1212 explanations were given; 214 out of 1212 ($\hat{p}$ = .177) fit at least one of the three hypothesised explanation types, 214 ($\hat{p}$ = .177) explanations were sufficient, 105 ($\hat{p}$ = .087) were compact, and 53 ($\hat{p}$ = .044) were minimal. A total of 998 out of 1212 ($\hat{p}$ = .823) explanations did not fit any of the three types. Again, the majority of explanations that did not fit any type consisted of a single argument (648).

We compared the proportions with which explanation types were selected for each AF to the baseline using one-proportion tests^5^ and found that they were all selected significantly more than both baselines (with *p* < .001 except for minimal explanations for $\mathcal{A}\mathcal{F}_2$ compared to both baselines, for which p = .007), except for sufficient explanations for $\mathcal{A}\mathcal{F}_2$ compared to the naive baseline ($\chi ^2$(1) = 2.35, *p* = .125).

### Additional Findings

After confirming our hypotheses, we conducted an additional exploratory analysis and found three interesting observations. First, participants generally select short explanations, meaning they consist of only a small number of arguments. Explanations can include anywhere from one to four arguments for $\mathcal{A}\mathcal{F}_1$, and one to five arguments for $\mathcal{A}\mathcal{F}_2$. The average length of explanations was 1.48 (SD = 0.54) for $\mathcal{A}\mathcal{F}_1$ and 1.74 (SD = 0.76) for $\mathcal{A}\mathcal{F}_2$. Participants most frequently provided explanations consisting of a single argument ($\hat{p}$ = .620 for $\mathcal{A}\mathcal{F}_1$ and $\hat{p}$ = .530 for$\mathcal{A}\mathcal{F}_2$). In most cases, these single-argument explanations consist of a related argument; the unrelated argument *I* is used infrequently compared to the other single-argument explanations. For $\mathcal{A}\mathcal{F}_1$, I is selected 86 out of 1196 times ($\hat{p}$ = .072), and for $\mathcal{A}\mathcal{F}_2$ 53 out of 1212 times ($\hat{p}$ = .043). Therefore, in the majority of cases, participants select a single related argument as an explanation.

Second, we found that participants more frequently provided explanations for the second AF that did not fit any of our explanation types, compared to the first AF. Therefore, we further investigated these explanations by dividing them into four categories: 1) explanations fully defending the topic, but including unrelated arguments, 2) explanations partially defending the topic, 3) explanations partially defending the topic and including unrelated arguments, and 4) only including unrelated arguments. The proportions of explanations that fit each of these categories can be found in Table 3. Most of the given explanations partially defended the topic and did not include unrelated information ($\hat{p}$ = .322 for $\mathcal{A}\mathcal{F}_1$, $\hat{p}$ = .672 for $\mathcal{A}\mathcal{F}_2$). For $\mathcal{A}\mathcal{F}_1$, explanations of this type were $\{E\}$ and $\{F\}$; both arguments defend the topic argument against one, but not both of its attackers. For $\mathcal{A}\mathcal{F}_2$, explanations partially defending the topic and not including unrelated arguments are $\{E,F,G\}$ and all one- and two-argument explanations not containing *I*. Out of these explanations, $\{F\}$ is most frequently selected (191 out of 1212), followed by $\{H\}$ (154 out of 1212), $\{G\}$ (141 out of 1212), and $\{E\}$ (141 out of 1112). Interestingly, explanation $\{G\}$ is used more than $\{E\}$, since these arguments were instantiated with the same content as for $\mathcal{A}\mathcal{F}_1$, for which both were approximately selected the same amount. The most frequently selected explanations $\{F\}$ and $\{H\}$ could be considered the most important arguments, because argument *F* defends the topic against two attackers and argument *H* defends the topic against an attacker that no other argument attacks. This means participants did identify what information they believed to be the most important in an explanation, but selected fewer arguments than needed to fully defend the topic.

Third, we investigated variance in explanation behaviour between participants, and we found some interesting patterns. About two-thirds of the participants (208 out of 301) varied the length of the explanations they chose; the other third of participants (92) always chose the same length explanation throughout the entire experiment. For all of these participants, the length they stuck with was one. Slightly over half of the participants (182) were consistent in their explanation length for at least one of the two AFs.

**Table 3:** Categories of explanations fitting none of the hypothesised types

	Total explanations that fit no type	1) Full defense, includes unrelated	2) Partial defense, no unrelated	3) Partial defense, includes unrelated	4) Only unrelated
$\mathcal{A}\mathcal{F}_1$	587 out of 1196 (.490)	79 (.066)	385 (.322)	37 (.031)	86 (.072)
$\mathcal{A}\mathcal{F}_2$	998 out of 1212 (.823)	20 (.017)	814 (.672)	111 (.092)	53 (.044)

Note. Four categories of explanations that fit none of the three hypothesised explanation types in this study, and the proportions of total explanations that fit these categories for both AFs. The proportions indicated for each of the four categories are based on the total explanations given for each AF and thus sum to the proportion of the first column

## Discussion

In this study, we investigated explanation preferences by comparing explanations provided by participants to three types of explanation definitions from computational argumentation: sufficient, compact, and minimal explanations. Participants selected explanations in argumentation-based scenarios based on two argumentation frameworks of differing complexity and spanning multiple domains. Each scenario consisted of a topic argument, counterarguments to the topic argument, arguments defending the topic by attacking those counterarguments, and one unrelated argument. Participants were instructed to construct an explanation for the topic argument, given the counterarguments, using the defending and unrelated arguments. We hypothesised that participants would prefer sufficient, compact, and minimal explanations more frequently than the two baselines, indicating a preference for these types of explanations over other possible explanations.

By comparing the explanations provided by participants to both the simulated and naive baselines described in Section “Main hypotheses”, we found support for our hypotheses. Participants selected explanations that fit the sufficient, compact, and minimal explanation types more frequently than expected based on two baselines, confirming our hypothesis that these types of explanations, as defined in [32], can align with human preferences. This preference should be interpreted with care. For $\mathcal{A}\mathcal{F}_2$, the majority of explanations did not fit the explanation types since participants generally gave shorter explanations than those that fit the explanation types. This is because in $\mathcal{A}\mathcal{F}_2$, a sufficient (minimal, compact) explanation needs to contain at least two arguments to successfully defend the topic argument against all its attackers *B*, *C*, and *D* (Fig. 2), so any short explanation consisting of just one argument will not fit any of the explanation types. Compared to the simulated baseline, which takes into account participants’ preference for short explanations, all three explanation types were selected more frequently than expected for both argumentation frameworks. Against the naive baseline, the pattern was more nuanced: in the smaller framework ($\mathcal{A}\mathcal{F}_1$), participants preferred all three explanation types, while in the larger framework ($\mathcal{A}\mathcal{F}_2$), only minimal and compact explanations were selected significantly more frequently than other explanations compared to the simulated baseline. The major difference between the two baselines is that the simulated baseline adjusted the expected proportions using the data collected in this study on explanation length. Therefore, this baseline controls for participants’ preference for short explanations and can show that, when we take this preference into account, participants’ explanation behaviour aligns with explanation types from formal argumentation literature.

Besides confirming our hypotheses, the most important finding is that participants preferred short explanations consisting of related arguments. These preferences were consistent across the two AFs. In the smaller argumentation framework ($\mathcal{A}\mathcal{F}_1$), the minimal explanation consisted of only one argument, and the compact explanations consisted of only two. For the larger AF ($\mathcal{A}\mathcal{F}_2$), the minimal and compact explanation consists of two and three arguments, respectively. Despite the increase in available information and the need for an additional argument to provide a minimal explanation, participants’ explanations were only 25 percent longer for $\mathcal{A}\mathcal{F}_2$ than for $\mathcal{A}\mathcal{F}_1$. This suggests that participants’ preference for short explanations remains relatively stable with an increase in the availability of information and the complexity of the situation. This is comparable to results by [34], where they found that participants employed simpler reasoning strategies by accepting fewer arguments for more complex AFs, showing that participants are more selective in complex cases. Explanations that did not fit any of the three tested types did, in most cases, consist of related arguments and partially defend the topic. Explanations that included unrelated arguments were rarely chosen, including explanations that are formally admissible (cf. Definition 1.1). This suggests that participants can identify and intentionally exclude unrelated arguments from explanations. These findings are consistent with [36], which also found a preference for explanations containing only directly related arguments, and [45], which found a preference for related arguments.

These findings about explanation length in combination with the found preference of participants for sufficient, minimal, and compact explanations may be explained through theories about the limitations of human cognition, such as *cognitive load theory* [39]. Cognitive load theory suggests that people have a maximum processing capacity; the load of processing multiple arguments and including them in explanations is considerable. Additional explanation length and unrelated elements cause unnecessary cognitive load; therefore, participants may include a similar amount of information for explanation in both AFs, even though more information is available for the second. For the second AF, more arguments are needed to provide a sufficient explanation; the additional load of needing to process and select more arguments could be a reason why many participants did not do so.

### Limitations and Future Work

This study identifies preferences for argumentation-based explanations, though the extent to which these findings generalise is subject to some limitations. These limitations fall into three main areas: (1) individual differences in behaviour, (2) differences in responses between scenarios, and (3) the setting of the experiment. This section further discusses these limitations, how they could be resolved, and what additional future work could be conducted.

While our main findings are based on aggregated results, we observed interesting differences between participants. One-third of participants were consistent in the number of arguments used in explanations, and over half were consistent within one AF. However, the other two-thirds of participants varied in explanation length depending on the scenario. Completion time varied widely between participants and was uncorrelated with explanation length: some participants quickly selected short explanations; others spent more time to arrive at similarly short explanations. These patterns may reflect unmeasured participant characteristics such as reasoning style or risk tolerance. This variability might limit our ability to generalise explanation preferences across the population. Further research into cognitive styles, decision strategies, or personality traits could explain these individual-level differences.

A second limitation of this study is the variation in responses to different scenarios. This was not fully unexpected, since we used a set of scenarios diverse in phrasing and context. Participant responses varied significantly across scenarios in the omnibus test, but no scenario was found to be significantly different in post-hoc tests. In prior work, on the Wason selection task [41] and argument evaluation [44], it has been found that participants may apply different reasoning strategies depending on context. In the current study, we focus on investigating human behaviour over a variety of scenarios. We conducted investigations into the effects of the context in which the scenarios were set and into the phrasing of the scenarios. However, due to a lack of statistical power in these post hoc investigations, we did not find any effects and did not further report on tests. Future work should investigate how context and phrasing influence reasoning to facilitate creating argumentation-based explanation definitions that can be used across diverse settings. In this regard, future work would benefit from applying established typologies of argument types, such as Walton’s argumentation schemes [56] and the systematic classifications proposed by Wagemans [57], to better account for variation in how different kinds of arguments are interpreted. More broadly, future work should investigate how context, wording, and domain knowledge of participants affect reasoning and explanation preferences.

Understanding how context and phrasing influence reasoning is essential for developing argumentation-based explanation frameworks that are robust across diverse settings. In this regard, future work would benefit from engaging more directly with established typologies of argument types, such as Walton’s argumentation schemes and the systematic classifications proposed by Wagemans, in order to better account for variation in how different kinds of arguments are interpreted. More broadly, future research should investigate how context, wording, argument type, and participants’ domain knowledge jointly affect reasoning and explanation preferences.

A third limitation to the generalizability of this study is the setting of the experiment. In our experiments stakes were low, and the time pressure was minimal. It is possible that in high-stakes or time-sensitive contexts, reasoning and explanation preferences are different. In time-sensitive contexts, explanation preferences might shift toward shorter or more selective reasoning, while in high-stakes settings, people might prefer longer explanations. Additionally, cognitive load may play a role in how participants select explanations. If participants in this study were limited in their explanation selection by cognitive capacity, reducing this might lead people to select longer explanations. For example, by simplifying individual arguments or by having participants evaluate arguments instead of generating them. Moreover, our participants were laypeople, whereas explanations of systems often serve domain experts whose explanation preferences may differ significantly from laypeople [58]. To understand how our results generalise to provide explanations to experts in high-stakes or time-sensitive settings, future studies should involve content specific to high-stakes domains and participants with relevant expertise.

We have discussed several limitations and opportunities for future work. To facilitate future work, we include 20 scenarios developed for this study, which can be used to represent two AFs and any of their subsets (see S3 Appendix). Future studies could address the generalizability issues mentioned previously. For example, by exploring whether experts in a domain have different explanations from non-experts and whether reducing cognitive load influences explanation preferences. If individual arguments are made shorter and easier to read, participants’ choices might change, suggesting that complexity plays a role in how explanations are selected.

Finally, the participant-generated explanations collected in this study can serve as a benchmark for evaluating argument-based explanation definitions. By comparing those explanation definitions to participant-generated explanations, researchers can assess the degree to which these explanation types align with human reasoning. This may support the development of more realistic and user-aligned explanation definitions, such as definitions that do not strictly require admissibility and interactive explanation definitions that use user preferences.

## Conclusion

In this study, we compared explanations provided by participants to three types of explanation definitions from computational argumentation: sufficient, compact, and minimal explanations. For the smaller argumentation framework, participants selected explanations that fit these types more frequently than expected under both baselines. For the larger framework, this pattern held for minimal and compact explanations across both baselines, showing that in these cases, participants’ explanation behaviour aligns with explanation types based on formal argumentation. However, for the larger argumentation framework, participants only showed a preference for sufficient explanations compared to the baseline that takes into account participants’ preference for short explanations, and not compared to the uniform baseline.

These findings show a correspondence between human explanatory behaviour and explanations based on computational argumentation, but also expose the limitations of existing formal frameworks. For the larger argumentation framework, the majority of explanations by participants were too short to be sufficient, minimal or compact according to definitions in computational argumentation, since these explanation types from [32] do not capture partial or non-admissible yet cognitively plausible explanations. To bring human and theoretical explanations closer together, future work should investigate how to create explanation definitions that allow for selective and potentially incomplete explanations, while preserving the guarantees of formal correctness. Together, this will contribute to the design of explanation definitions that are both formally sound and psychologically realistic. By sharing our experimental setup, including scenarios and argumentation frameworks that can be used by others (see S3 Appendix), we hope to support further empirical research into argumentation-based explanations.

## Supplementary Information

Below is the link to the electronic supplementary material.

Supplementary file 1 (pdf 75 KB)

Supplementary file 2 (pdf 62 KB)

Supplementary file 3 (pdf 71 KB)

Supplementary file 4 (pdf 78 KB)

## Data Availability

All study materials are openly accessible via the OSF repository linked in this manuscript. In accordance with Utrecht University’s data management policy, the collected data are securely stored and can be made available upon reasonable request to the author.

## Footnotes

1 In the instructions to participants, we use “true” as shorthand for an argument which is acceptable given the other arguments in the AF.

2 The expected counts for some cells was less than five, therefore, the Chi-square test could not be used.

3 For $\mathcal{A}\mathcal{F}_1$, Cramér’s V = .286, for $\mathcal{A}\mathcal{F}_2$, Cramér’s V = 0.277.

4 This test was used because the difference between explanation lengths for$\mathcal{A}\mathcal{F}_1$and$\mathcal{A}\mathcal{F}_2$per participant were found to be not be normally distributed using a Shapiro-Wilk test for normality (W=0.96, *p* < 0.001) $\mathcal{A}\mathcal{F}_1$and $\mathcal{A}\mathcal{F}_2$per participant were found to be not be normally distributed using a Shapiro-Wilk test for normality (W=0.96, *p* < 0.001)

5 Assumptions for this test were met: trials were independent, and both the number of expected successes and expected failures exceeded 5 in all cases, satisfying the normal approximation to the binomial distribution.
