# Supplementary material for: Empirically Testing Explanation Preferences in Computational Argumentation
Source: Cognit Comput. 2026 Sep 12;18(1):109. doi: 10.1007/s12559-026-10654-y (PMC13570894; doi:10.1007/s12559-026-10654-y)
Supplement: Supplementary file 1 [file 12559_2026_10654_MOESM1_ESM.pdf]

S1 Appendix. Consent and instructions.

Welcome to the Study 'Explanation based on Formal Argumentation'
By Roos Scheffers, Graduate School of Natural Sciences, Utrecht University.

1. Introduction

My name is Roos, and I am a graduate student at Utrecht University. I'm conducting a study on explanations in the field of explainable artificial intelligence as part of my master's thesis under the supervision of Floris Bex. You are invited to take part in this study. Before you decide, it is important to understand why the research is being done and what it will involve.

2. About the study

Models created using artificial intelligence can be very complex. The field of explainable artificial intelligence aims to explain these very complex models and to create functional models with reduced complexity. To be able to explain models to people, it is important to understand what explanations people prefer and use. This study will collect such data and compare it to theories of explanation based on formal argumentation.

3. Your rights

Participation is voluntary. Your data for this study will only be collected if you consent to this. If you decide not to participate, you do not have to take any further action. You do not need to sign anything. Nor are you required to explain why you do not want to participate. If you decide to participate, you can always change your mind and stop participating at any time, including during the study. You will even be able to withdraw your consent after you have participated. However, if you choose to do so, we will not be required to undo the processing of your data that has taken place up until that time. The personal data we have obtained from you up until the time when you withdraw your consent will be erased (where personal data is any data that can be linked to you, so this excludes any already anonymised data)

4. Approval of this study

This study has been allowed to proceed by the Research Institute of Information and Computing Sciences based on an Ethics and Privacy Quick Scan. If you have a complaint about the way this study is carried out, please send an email to: ics-ethics@uu.nl. If you have any complaints or questions about the processing of personal data, please send an email to the Faculty of Sciences Privacy Officer: privacy-beta@uu.nl. The Privacy Officer will also be able to assist you in exercising the rights you have under the GDPR. For details of our legal basis for using personal data and the rights you have over your data, please see the University's privacy information at www.uu.nl/en/organisation/privacy. If you have any questions or concerns about this research, please contact Roos Scheffers at r.j.scheffers@uu.nl.

Please read the statements below and tick the final box to confirm you have read and understood the statements, and upon doing so, agree to participate in the project.

- I confirm that I am 18 years of age or over.
- I confirm that the research project "Properties of Argumentation-based Explanations" has been explained to me.
- I consent to the material I contribute being used to generate insights for the research project.
- I understand that my participation in this research is voluntary and that I may withdraw from the study at any time without providing a reason, and that if I withdraw, any personal data already collected from me will be erased.
- I consent to allow the fully anonymized data to be used in future publications and other scholarly means of disseminating the findings from the research project.

- I understand that the data acquired will be securely stored by researchers, but that appropriately anonymized data may in future be made available to others for research purposes.
- I understand that the University may publish appropriately anonymized data in appropriate data repositories for verification purposes and to make it accessible to researchers and other research users.

Study instructions

Please carefully read the following instructions.

In this study, you will be presented with *8* scenarios, each of which consists of several arguments and counterarguments. First, you will see the *topic argument*, which you can always assume to be true. Below the topic argument, you will see several *counterarguments* to the topic. Below the counterarguments are several other arguments. Your task will be to explain why the conclusion of the topic argument is the case, using the other arguments in the scenario. Choose the options that you feel explain the topic argument's conclusion best, by checking the box next to the arguments. You can select any number of arguments to explain the topic argument.

For this study, I'm interested in how you personally would choose to explain the topic. There are no right or wrong answers; you can explain each scenario however you feel is best. Please select the options that best correspond to how you would explain the topic argument.

There is no time limit on this task, so feel free to take as long as you need to read all the arguments carefully. Every question will include an info button that you can click to see a shortened version of this explanation. Feel free to take a look at the first question and go back to the instructions if you want to read them again. You can go back to these instructions at any time.
