# Supplementary material for: Empirically Testing Explanation Preferences in Computational Argumentation
Source: Cognit Comput. 2026 Sep 12;18(1):109. doi: 10.1007/s12559-026-10654-y (PMC13570894; doi:10.1007/s12559-026-10654-y)
Supplement: Supplementary file 2 [file 12559_2026_10654_MOESM2_ESM.pdf]

S2 Appendix. Experiment layout.

Claim: Jessie saw Stephen at the crime scene yesterday at the time of the crime. Therefore, Stephen is guilty.
However, Stephen's father testifies that Stephen was having dinner with his family at the time of the crime. Therefore, Stephen couldn't have been seen at the crime scene at the time of the crime. *And* Stephen testifies that he has never been to the crime scene. Therefore, Stephen couldn't have been seen at the crime scene at the time of the crime.
And it was night and dark outside at the time of the crime, so Jessie could have mistakenly thought the perpetrator was Stephen.

- ☐ Stephen's father can be very forgetful sometimes, so he might have misremembered having dinner with Stephen.
- ☐ Neither Stephen nor his father are reliable witnesses on Stephen's actions; therefore, their testimonies cannot be trusted.
- ☐ There is a picture of Stephen at the crime scene two years ago, so Stephen is lying when he says he's never been at the crime scene.
- ☐ The area around the crime scene is generally well lit at night, so it shouldn't be an issue to recognise someone.

Claim: AI system X is biased and makes unfair decisions. Therefore, AI system X is unethical. *However*, developer 1 claims that AI system X was trained on diverse datasets. Therefore, it cannot be biased. *And* developer 2 testifies that the algorithm of AI system X is designed to eliminate biases. Therefore, it cannot make unfair decisions. *And* an independent auditor reports that the data used by AI system X is very representative of the population. Therefore, it's not biased.

- ☐ Developer 1 has previously ignored issues of data bias, so their claim might not be reliable.
- ☐ Both developers are part of the team that created AI system X, and hence might be biased in defending it.
- ☐ A study found that AI system X's decisions disproportionately affect certain groups. Therefore, the system's fairness is questionable.
- ☐ The independent auditor's report might have overlooked recent updates to the dataset and algorithm. Therefore, their conclusion might be outdated.
- ☐ Developer 3 is an expert in auditing AI systems and can therefore be trusted to identify bias in systems
