# Supplementary material for: Empirically Testing Explanation Preferences in Computational Argumentation
Source: Cognit Comput. 2026 Sep 12;18(1):109. doi: 10.1007/s12559-026-10654-y (PMC13570894; doi:10.1007/s12559-026-10654-y)
Supplement: Supplementary file 3 [file 12559_2026_10654_MOESM3_ESM.pdf]

S3 Appendix. Scenarios.

Scenario 1

(A) Jessie saw Stephen at the crime scene yesterday at the time of the crime. Therefore, Stephen is guilty.

(B) Stephen's father says that Stephen was having dinner with his family at the time of the crime. Therefore, Stephen couldn't have been at the crime scene at the time of the crime.

(C) Stephen says that he has never been to the crime scene. Therefore, Stephen couldn't have been seen at the crime scene at the time of the crime.

(D) It was night and dark outside at the time of the crime, so Jessie was wrong in thinking he saw Stephen.

(E) Stephen's father is very forgetful, so he misremembered having dinner with Stephen.

(F) Neither Stephen nor his father are reliable sources of Stephen's actions. Therefore, their claims cannot be trusted.

(G) There is a picture of Stephen at the crime scene two years ago, so Stephen is lying when he says he's never been to the crime scene.

(H) The area around the crime scene is generally well-lit at night, so it wasn't dark around the crime scene at the time of the crime.

(I) Jessie's credit card was used to make a purchase near the crime scene around the time of the crime. Therefore, it is likely Jessie was at the crime scene when the crime occurred.

Scenario 2

(A) A user says the software crashes when they click the "Save" button. So, there is a problem with the software.

(B) Developer A says they tested the "Save" button, and it works fine on their computer. So, there is no problem.

(C) Developer B looked at the code and found no issues with the "Save" button. So, there is no problem.

(D) The software was running on an older version of the operating system on the user's computer. So, the crash happened because of compatibility issues, not because of a problem with the software.

(E) Developer A forgot to update their test setup. So, their test results are not up-to-date.

(F) Developers A and B are using a newer version of the software than the user. So, their tests are different from what the user is seeing.

(G) Developer B missed some special cases. So, their code check is not complete.

(H) Other users are using the software on older operating systems. So, the old version does not cause compatibility problems.

(I) Reports take at least two weeks to be processed. So, the developers are behind schedule.

Scenario 3

(A) System X uses biased data and makes unfair decisions. So, system X is not ethical.

(B) Developer 1 says that system X was made with different types of data. So, it cannot be unfair.

(C) Developer 2 says system X is made to remove unfairness. So, it cannot make unfair decisions.

(D) An inspector reports that the data used by AI system X represents the population well. So, it is not biased.

(E) A study reports that the datasets system X was made with are very similar. So, the system was not trained on different types of data.

(F) A senior developer says system X was made quickly. So, it was not trained on many different datasets or designed to remove unfairness.

(G) A study found that AI system X's decisions affect some groups more than others. So, the system is not fair.
(H) The inspector missed some changes in the data and system. So, their report is not trustworthy.
(I) Developer 2 says AI system Y was made with unfair data. So, system Y is not fair.

Scenario 4

(A) Scientist A's data shows that global temperatures have gone up in the last 100 years, so climate change is real.
(B) Scientist B says that the data is affected by heat from cities, and does not reflect what is happening everywhere. So, the data is misleading.
(C) Opponent C says that climate data is not reliable and has many gaps. So, we cannot trust results based on it.
(D) Opponent D says that natural changes, like solar cycles, can also explain the rise in global temperatures. So, the data from Scientist A does not prove that climate change is real.
(E) New methods have been created to fix the problem of city heat in climate data. So, Scientist B's idea is outdated.
(F) Both Scientist B and Opponent C are paid by groups that make money from fossil fuels. So, their opinions may be biased.
(G) New satellite data supports the rise in global temperatures that the data showed. So, the data is not misleading.
(H) Scientist F says the rise in global temperatures is more connected to pollution from human activities than to natural changes. So, natural changes cannot explain the rise in temperature.
(I) A new satellite was launched last week to make climate data better. So, future climate data will be more accurate.

Scenario 5

(A) The judges decided that contestant A's volcano experiment was the best project at the science fair because it clearly showed a chemical reaction.
(B) Contestant B says that the volcano project uses everyday materials and is not very original. So, it should not be the best.
(C) A science teacher says the chemical reaction in the volcano experiment is simple and does not show advanced knowledge. So, it is not the best experiment.
(D) An observer says that contestant A's experiment does not connect to real-world problems, which is important in experiments. So, it is not the best project.
(E) Contestant A explains that they used a new mix of materials that are not usually used in volcano experiments, so the project is original.
(F) The audience at the fair agreed that the materials and the method show creativity and good scientific understanding, proving the project is not too simple or unoriginal.
(G) Before the experiment, contestant A wrote a detailed report about it. So, they have shown a strong understanding of the experiment.
(H) The rules of the science fair say that projects should be educational for children. So, the experiments don't need to relate to real-world problems.
(I) The audience liked contestant C's project. So, contestant C's project is the audience's favorite.

Scenario 6

(A) The CEO says that expanding into the Asian market will help the company make more money.
(B) The CFO thinks the Asian market already has too many competitors, so it will be hard for new companies to succeed.
(C) A market analyst reports that what customers in Asia want is different from what our products offer, so the products may not sell well there.

(D) A logistics manager says that shipping costs and taxes will lower profits for products sold in Asia.
(E) The regional manager in Europe points out that the company has been successful in similar markets and thinks small changes can help with the competition in Asia.
(F) The business development team mentions that the company has a history of overcoming challenges like new customer wishes and competition.
(G) Surveys done by the company show that customers in nearby regions are more interested in our products, which could mean success in Asia too.
(H) The logistics manager is not aware of recent trade deals, so they cannot accurately predict the costs of taxes.
(I) A trade fair showing new Asian technology is planned for next month. So, many interested people will be there.

Scenario

(A) A policymaker suggests a new law to increase financial help for solar energy, saying it will help fight climate change.
(B) An opposing lawmaker says the subsidies are too expensive and will hurt the national budget.
(C) An environmental scientist says focusing only on solar energy won't be enough to meet the country's energy needs.
(D) An economic analyst is worried that this money will take away funds needed for other important environmental projects.
(E) The CEO of a solar company says the price of solar panels has gone down, making them a cheaper option.
(F) A renewable energy expert says that the lawmaker and the scientist are not counting on the long-term savings from using solar energy.
(G) New technology has made solar energy more efficient, which goes against the scientist's idea that solar power won't be enough.
(H) A financial expert shows data from other countries where similar subsidy programs worked well, so it will not take money away from other projects.
(I) A recent survey shows that public support for wind energy subsidies is at its highest. So, supporting this could help politicians get more votes.

Scenario 8

(A) Reviewer 1 says that the paper makes an important contribution to the field. So, the paper should be accepted.
(B) Reviewer 2 says that the paper does not have enough experimental proof. So, the paper should be rejected.
(C) Reviewer 3 argues that the paper's theory has problems. So, the paper should be rejected.
(D) The paper does not follow the journal's style, showing a lack of care. So, the paper should be rejected.
(E) Reviewer 2 is known to be very critical. So, their negative review is biased.
(F) The paper has an appendix with detailed experimental proof and an explanation of the theory. So, these parts are not lacking or flawed.
(G) Reviewer 3 often publishes work that challenges other theories. So, their review is biased by their research.
(H) Papers do not need to follow the journal's style during the review. So, the style does not show a lack of care.
(I) The journal editor shared new rules for submitting papers to improve the review process. So, future reviews will be faster.

Scenario 9

(A) Doctor A says the patient has Disease X based on their symptoms. So, the patient has Disease X.

(B) Doctor B says the symptoms show the patient has Disease Y. So, the diagnosis of Disease X is wrong.

(C) The patient's family has a history of Disease Y. So, the patient has Disease Y.

(D) Doctor A has made mistakes in diagnosing similar cases before. So, their diagnosis cannot be trusted.

(E) Doctor B is an expert in rare diseases and is focusing too much on the chance of Disease Y. So, their opinion is biased.

(F) Lab tests do not show any signs of Disease Y. So, the patient does not have Disease Y.

(G) Disease Y is not passed down in families. So, the patient's family history of Disease Y does not matter.

(H) In the cases where Doctor A made mistakes, the symptoms were not as clear as in this case. So, Doctor A has not made the same mistake in similar cases.

(I) Patient surveys show that people are unhappy with Doctor C's diagnosis. So, Doctor C's methods should be checked.

Scenario 10

(A) In a book review, Critic A says the main character's actions are driven by a desire for revenge. So, the theme of the book is revenge.

(B) Critic B says the main character's actions are not about revenge, but about seeking justice. So, the theme of the book is justice.

(C) Other interpretations suggest that the main character's actions are complicated and cannot be put into just one category. So, the theme does not fit any single category.

(D) Literary analysis of the book shows that the main theme is justice. So, the theme is not revenge.

(E) Critic B has a background in law. So, their focus on justice is biased.

(F) Critic A has written a lot about revenge in literature. So, their argument is likely better supported than Critic B's or the alternative interpretations.

(G) The complicated actions of the main character make the theme deeper, not unclear. So, the theme can still be understood as a single category.

(H) Literary analysis is open to different opinions. So, it cannot give a final answer on what the theme of the book is.

(I) The antagonist does not appear until the second half of the book. So, they do not affect the themes in the first half.

Scenario 11

(A) Employee A says that Manager X favors certain employees by giving them better treatment. So, Manager X is not a good manager.

(B) Manager X says they reward employees who do very well. So, the treatment is based on performance, not favoritism.

(C) HR records show that all employees under Manager X have similar performance reviews. So, on the records, Manager X does not show favoritism.

(D) Employee A has had problems with Manager X in the past. So, Employee A is not a reliable source about Manager X.

(E) Rewards based on performance should be clear and fair. If not, special treatment is still favoritism.

(F) It would be bad for both Manager X and HR if favoritism is proven. So, they are not reliable sources of information.

(G) Performance reviews are based on opinions and can be biased. So, HR records do not give a definite picture of performance.

(H) Several other employees have reported concerns about favoritism, too. So, Employee A's claim is not biased.

(I) Manager Y is friends with Manager X. So, Manager Y is not a reliable source of information about Manager X.

Scenario 12

(A) Teacher A suggests adding a computer science class to the curriculum to help students get ready for future jobs. So, the course should be added.

(B) Parent B says the current curriculum is already too hard, and adding another class will stress the students. So, the course should not be added.

(C) Surveys show that most students feel they already have a lot of work. So, the course should not be added.

(D) After-school programs can offer computer science without adding more to students' workload. So, the course doesn't need to be part of the regular curriculum.

(E) Parent B is mostly worried about their own child's workload. So, their argument is biased.

(F) The workload at this school is less than at other schools in the area. So, Parent B and the students are wrong when they say the workload is too high.

(G) Students often feel their school workload is high. So, the surveys are more negative than the reality.

(H) After-school programs do not include all students. So, they are not the best way to teach computer science to everyone.

(I) The school recently improved its technology. So, their online classes are better now.

Scenario 13

(A) The patient has a high fever and a bad cough, which are signs of a lung infection. So, the patient has a lung infection.

(B) Doctor A says that the patient's lungs sound normal during the exam. So, the patient does not have a lung infection.

(C) Doctor B says the chest X-ray shows no signs of pneumonia. So, the patient does not have a lung infection.

(D) The patient was recently near someone with the flu. So, the symptoms are likely from the flu, not a lung infection.

(E) Doctor A has missed lung infections before. So, their exam is not reliable.

(F) Both Doctor A and Doctor B have not thought about the full range of diseases that could cause these symptoms. So, their diagnoses are incomplete.

(G) The patient has had chronic bronchitis before, which makes it harder to understand the X-ray. So, Doctor B's conclusion is wrong.

(H) The patient's white blood cell count is high, which is common in infections and not common in the flu. So, the patient does not have the flu.

(I) A health campaign has started to teach people how to prevent the flu. So, there will be fewer flu patients in the future.

Scenario 14

(A) The coach says that player X should be in the starting lineup because they have played very well in recent games. So, player X should start.

(B) The assistant coach says that player X has a small injury, and starting could make it worse. So, player X should not start.
(C) Player Y says that they are in better shape than player X. So, player Y should start instead.
(D) The team's sports analyst says that player X has a history of not playing well in important games. So, player X is not a good choice for the starting lineup.
(E) The assistant coach is exaggerating the injury problem. So, their argument is not correct.
(F) Both the assistant coach and player Y are biased because they are close friends. So, their arguments are affected by personal interests.
(G) Recent fitness tests show that player X is in good shape. So, player Y is not in better form than player X.
(H) The sports analyst made a mistake when judging the current star player. So, their opinions are not reliable.
(I) The team's morale has gotten much better since the new training methods were introduced. So, the new methods were a good idea.

Scenario 15

(A) Botanist A says that the plant is species X because of its flowers. So, the plant is species X.
(B) Botanist B says that the leaves do not look like those of species X. So, the plant is not species X.
(C) Botanist C says the plant lives in a habitat that is normal for species Y, not species X. So, the plant is species Y.
(D) The plant is a mix of both species X and Y. So, it is a new species that is not X or Y.
(E) Botanist B has made mistakes before by focusing too much on the leaves. So, their argument is too focused on leaf shape and not reliable.
(F) Both Botanist B and Botanist C have done a lot of research on species Y. So, their opinions are biased because of their past work.
(G) Habitat is not the only way to identify a plant, as many plants can live in different environments. So, the plant can still be species X even if it is found in a habitat typical for species Y.
(H) Botanist D says the plant's DNA only consists of one species. So, the plant is not a mix of species X and Y.
(I) Botanist D is an expert on species Y, so they can be trusted to identify a plant as being species Y.

Scenario 16

(A) Alice says that the movie Journey's End has high ratings and has won many awards. So, Journey's End is the best movie to watch with their friends.
(B) Bob says that Star Adventures has great visual effects and that ratings don't show how fun a movie is. So, Star Adventures is the best movie to watch.
(C) Clara thinks Journey's End is boring and slow, so it is not for everyone.
(D) Dave believes the group likes comedies more than dramas like Journey's End, so it's not a good choice.
(E) Emma says that Bob loves science fiction, so he is biased toward science fiction movies.
(F) Felix says that Bob and Clara might not be seeing how good the story in Journey's End is.
(G) Grace argues that Clara doesn't like award-winning movies, which makes her biased against Journey's End.
(H) Henry says that even though Dave usually likes comedies, he has rated dramas highly before, which means a drama could still be a good choice.
(I) Henry and Grace have the same favorite movie, so their tastes are alike.

Scenario 17

(A) Professor Y says that the newly discovered species has feathers and hollow bones, so it should be classified as a bird.

(B) Student A argues that the species has teeth, which is not common in modern birds. So, the species should not be classified as a bird.

(C) Student B says the species has a urinary bladder, something birds do not have. So, it should not be classified as a bird.

(D) A zoologist points out that the species does not have a wishbone, a bone that all birds have. So, the species is not a bird.

(E) An ornithologist criticizes Student A for ignoring the fact that ancient birds had teeth, showing that having teeth does not mean it's not a bird.

(F) Professor Y questions both students' arguments, saying they do not have much experience in the field.

(G) A paleontologist disagrees with Student B because they only studied one specimen, so they cannot be sure if all birds of this species had a bladder.

(H) A biologist says the skeleton study of the species was not complete. So, we cannot be sure if the species had a wishbone or not.

(I) Next month, a biology conference will discuss the effects of climate change on global biodiversity. So, the discovery of a new species is important and could be presented at the conference.

Scenario 18

(A) Research shows that students are not doing well in public schools. So, the education system needs to change.

(B) Teacher A says the problem is not the education system but the lack of parents being involved. So, no changes are needed.

(C) Teacher B says that the curriculum is outdated, but the education system itself is fine. So, the education system does not need to change.

(D) A local government report says the issue is not the education system, but a lack of funding. So, changing the system won't fix the problem.

(E) Teacher A has been criticized for their old-fashioned teaching methods, which affect their opinion on updating the education system.

(F) Critics say teachers do not know what is happening in other schools. So, the lack of parental involvement and an outdated curriculum are only part of the problem, not the full issue.

(G) Some big changes were made to the curriculum two years ago. So, Teacher B is wrong in saying the curriculum is outdated.

(H) Studies from nearby areas with similar funding show better student performance after updating their education systems. So, the problem is not just about money or poor infrastructure.

(I) The education board is thinking about extending school hours to offer more time for extra courses. So, more classes can be added.

Scenario 19

(A) Meteorologist A sees no clouds in the weather images. So, they believe it will be sunny tomorrow.

(B) Meteorologist B says that that is not enough information to predict a sunny day. So, Meteorologist A cannot know if it will be sunny.

(C) Meteorologist C says that the high temperature could lead to a storm tomorrow afternoon. So, it won't be sunny.

(D) A climate expert thinks global warming is making weather predictions less reliable. So, the prediction for tomorrow is not correct.

(E) Colleagues point out that newer models than the one Meteorologist B uses can predict the weather based on cloud patterns. So, there is enough information to predict a sunny day.

(F) Observations show that Meteorologists B and C may pay too much attention to uncertain information. So, their claims are not reliable.

(G) Meteorologist C has paid too much attention to temperature before. So, their opinion based on temperature is wrong.

(H) Meteorologist D says global warming does not affect short-term weather predictions. So, the prediction for tomorrow is not affected by global warming.

(I) Local weather stations are upgrading their equipment to make predictions better, so local weather predictions will improve.

Scenario 20

(A) Alex suggests watching a horror movie because horror movies are exciting and fun. So, a horror movie is the best choice for movie night.

(B) Bob argues that horror movies can cause nightmares, so they are not a good choice for a fun evening. So, they should not watch a horror movie.

(C) Cara says that not everyone finds horror movies exciting, and some people find them stressful. So, horror movies are a bad choice for movie night.

(D) A recent poll in their group of friends shows that most prefer comedy over horror. So, it is better to watch a comedy movie.

(E) Bob doesn't like horror movies because he had a bad experience with one movie. So, a different horror movie might be okay to watch.

(F) Bob and Cara recently had an argument with Alex, so they may be disagreeing with Alex's movie choice because of that.

(G) Cara doesn't usually like popular movies. So, she is not the best judge of what others would enjoy.

(H) Not everyone in the friend group took part in the poll. So, the poll does not show what the whole group wants.

(I) A study shows that different people experience drama films in different ways depending on their age. So, people feel differently about drama movies.
