# Supplementary material for: Empirically Testing Explanation Preferences in Computational Argumentation
Source: Cognit Comput. 2026 Sep 12;18(1):109. doi: 10.1007/s12559-026-10654-y (PMC13570894; doi:10.1007/s12559-026-10654-y)
Supplement: Supplementary file 4 [file 12559_2026_10654_MOESM4_ESM.pdf]

S4 Appendix. Scenario creation.

This appendix provides further details on how the scenarios used in the study were constructed and why specific design choices were made.

For this study, we created 20 scenarios to instantiate two abstract argumentation frameworks. This approach follows prior work on empirical studies of abstract argumentation(e.g. [1, 2]), where multiple natural-language instantiations of AF are presented to participants in a task.

An important consideration in the design of the scenarios was the large number of factors that can influence how natural-language arguments are interpreted. These include, for example, the information contained in arguments, the types of attacks between arguments, the topics of the scenarios, and the phrasing used in the arguments. Given this wide range of potential sources of variation, we did not attempt to control each factor individually. Instead, we intentionally incorporated variation in the formulation and content of arguments across scenarios. This design choice supports the aim of this study to test whether hypotheses hold across diverse scenarios, rather than being tied to specific phrasings or contexts.

At the same time, this design choice comes with an inherent limitation: although the scenarios are structurally equivalent from the perspective of abstract argumentation, they are not necessarily interpreted as such by participants. As a result, some variation in participant responses across scenarios is to be expected. We did take several measures to reduce this variation in responses:

- **Standardized presentation.** The presentation of scenarios to participants was standardized (see S2 Appendix). In each scenario, the topic argument was introduced first, and participants were instructed to assume it to be true. The arguments that attack the topic argument are then explicitly presented as counterarguments. The remaining arguments, consisting of defenders of the topic argument and unrelated arguments, are subsequently listed as 'other arguments', from which participants can select explanations, that is, possible defenders of the topic argument. By explicitly identifying counterarguments and indicating the direction of attack, the design reduces ambiguity about how the arguments relate to one another. Additionally, it ensures that the arguments that need to be countered in order to defend the topic argument are explicitly introduced.
- **Grounding in prior work.** Prior research shows that participants can reliably identify defending arguments, even when arguments and attacks vary in phrasing and type [3, 4]. The formulation of our scenarios was therefore informed by earlier work in which participants successfully recognized the relation between arguments in natural language [1, 2, 5].
- **Topic diversity.** Scenarios covered a variety of topics in which arguments and disagreements might happen. The three main themes among the topics are business, science, and entertainment. Participants were probably familiar with these topics, and they are written in such a way that no prior knowledge is needed to understand the scenarios.

References

[1] Iyad Rahwan, Mohammed I. Madakkatel, Jean-François Bonnefon, Ruqiyabi N. Awan, and Sherief Abdallah. Behavioral Experiments for Assessing the Abstract Argumentation Semantics of Reinstatement. *Cognitive Science*, 34(8):1483–1502, 2010.

[2] Mathieu Guillaume, Marcos Cramer, Leendert van der Torre, and Christine Schiltz. Reasoning on conflicting information: An empirical study of Formal Argumentation. *PLOS ONE*, 17(8):e0273225, 2022. Publisher: Public Library of Science.

[3] Roos Scheffers, Floris Bex, and AnneMarie Borg. Related Explanations in Formal Argumentation, an Empirical Study. In *Computational Models of Argument*, pages 265–276. IOS Press, 2024.

[4] Predrag Teovanović, Srdjan Vesic, and Bruno Yun. Human Compliance with Computational Argumentation Principles. *Artificial Intelligence*, page 104457, November 2025.

[5] Marcos Cramer and Mathieu Guillaume. Directionality of Attacks in Natural Language Argumentation. In Claudia Schon, editor, *Proceedings of the fourth Workshop on Bridging the Gap between Human and Automated Reasoning*, volume 2261 of *CEUR Workshop Proceedings*, pages 40–46, Stockholm, Schweden, 2018. CEUR. ISSN: 1613-0073.
